# Evaluation of efficacy of GCSF in reducing neutropenia among carcinoma patients undergoing anti-cancer chemotherapy. A prospective cohort study

**DOI:** 10.1371/journal.pone.0315435

**Published:** 2025-01-02

**Authors:** Maria Kakar, Sami Ullah, Amjad Khan, Shabnam Nazir

**Affiliations:** 1 Department of Pharmacy, University of Peshawar, Peshawar, Pakistan; 2 Department of Pharmacy, Kohat University of Science and Technology (KUST), Kohat, Pakistan; Waseda University: Waseda Daigaku, JAPAN

## Abstract

The use of granulocyte colony-stimulating factor (GCSF) to control febrile neutropenia (FN) caused by anti-cancer chemotherapy is well documented but it still needs to evaluated with respect to the specific type of cancer and chemotherapeutic agents. The present study evaluates the efficacy of adjunctive GCSF for treating FN after taking anticancer therapy by measuring clinical, hematological and microbiological outcomes. It is a single center study conducted at Hayatabad Medical Complex (HMC), Peshawar, Pakistan. Adult patients of both genders, suffering from different types of sarcomas and taking anticancer chemotherapy were included in the study. The study was conducted between January 2023 and January 2024. Baseline data including demographic data, medication history and hematological evaluation of all the patients was recorded at the time of enrolment. Primary outcomes of the study were the extent of absolute neutrophil count (ANC) recovery, duration and severity of neutropenia (grade IV), period to fever resolution. After the therapy (with and without adjunctive GCSF) clinical outcomes, hematological evaluation and microbiological data was compared and evaluated. All the data was statistically analyzed by SPSS (IBMS, version 20). A total number of 120 patients were investigated out of which data of 109 patients was included. Out of 109 patients, 64 (58.72%) received adjunctive GCSF therapy, and 45 (41.28%) did not receive adjunctive GCSF. Comparison of the data showed that the patients receiving adjunctive GCSF had a significant improvement ANC recovery time, better recovery of fever and patients were free of infections. This study concluded that adjunctive GCSF therapy benefits the patients undergoing anticancer treatment for different types of carcinoma.

## Introduction

Development of chemotactic and phagocytic defects along with a decline in neutrophil count is common with anti-cancer treatment [1]. Febrile neutropenia (FN) is a medical disorder developing in cancer patients getting strong anticancer chemotherapeutic agents. FN is said to be a single episode of fever higher than 38.3°C, and absolute neutrophil count (ANC) of <1.0 x 10^9^/L up to <0.5 x 10^9^/L [2]. The deadline for a less ANC nadir is 0.1×10^9^/L, whereas ANC recovery time is the time from anticancer treatment until the patient’s ANC increased to 2 × 10^9^/L, after the expected nadir [3,4]. FN can cause severe adverse effects [5], and a rapid medical attention is essential because of decreased immunity. Pus, abscesses, and infiltrates on chest X-ray are the distinctive symptoms of infections, and all these symptoms subside with recovery of neutrophil count [6]. FN can be well managed by initiating empiric therapy with broad spectrum antibiotics and supportive care as soon as fever appears. No matter how well FN is being managed still death rate is 10% among FN patients experiencing infections. Hence, FN is the foremost menace to patients given anticancer therapy, consequently affecting quality of life, with higher infection risk and even death [7].

Chemotherapy induced FN may also cause dose interruptions and even sometimes stoppage of chemotherapy, which adversely affects treatment outcomes. Effects of neutropenia leading to increased risk of infection in cancer patients was first documented in the mid of 1960s. In a study, patients having ANC less than 1.0×10^9^/L for 7 days had more than 50% probability of developing an infection and the risk of infection approached 100% as the neutropenia prolonged. Order of ANC severity is categorized [8,9] as;

**Grade-1:** ANC of 1500 cells/mm^3^

**Grade-2:** ANC between 1000–1500 cells/mm^3^

**Grade-3:** ANC between 500–1000 cells/mm^3^

**Grade-4:** ANC less than 500 cells/mm^3^

Granulocyte colony stimulating factor (GCSF) increases the proliferation and differentiation of granulocyte macrophage colony stimulating factors (GM-CSF). Reduction in the duration of neutropenia and FN has been proved by growth factors [10,11]. GCSF has been recommended for preventing FN by different legal and professional clinical bodies [11]. However, its efficacy for treating FN is debatable because different clinical trials and meta-analysis have reported contradictory results. There was no significant reduction in the hospital stay in two studies, whereas another study showed 1-day reduction in hospital period [12]. Furthermore, any improvement in clinical outcomes like decline in neutropenia length and upgrading of neutrophil recovery have not been consistent [13]. Consequently, unless and until a patient is at a higher risk of infections or shows worse prognostic factors predisposing them to bad clinical outcomes i.e. lengthy hospital stay or death, existing guidelines do not approve of a regular use of adjunctive GCSF. To identify such patients different clinical predictive models have been developed.

In the light of these facts, present study was intended to estimate the effectiveness of adjunctive GCSF in treating FN caused by anticancer chemotherapy [14]. Likewise, founding the patients types who would get benefit from concomitant GCSF will permit oncologists to make decision of GCSF therapy.

## Methodology

### Design of the study

This was a prospective cohort study conducted at the Hayatabad Medical Complex (HMC), Peshawar, Pakistan, from January 2023 to January 2024. This study was approved by the “Committee for Ethics in Research” Department of Pharmacy, University of Peshawar, and was endorsed by the “Ethical Review Board” Hayatabad Medical Complex (HMC), Peshawar, Pakistan. Detailed timeline of the study is;

- Ethical approval of the study: September 6, 2022.
- Recruitment: January 2023
- Completion of the study: January 2024

**Fig 1:**
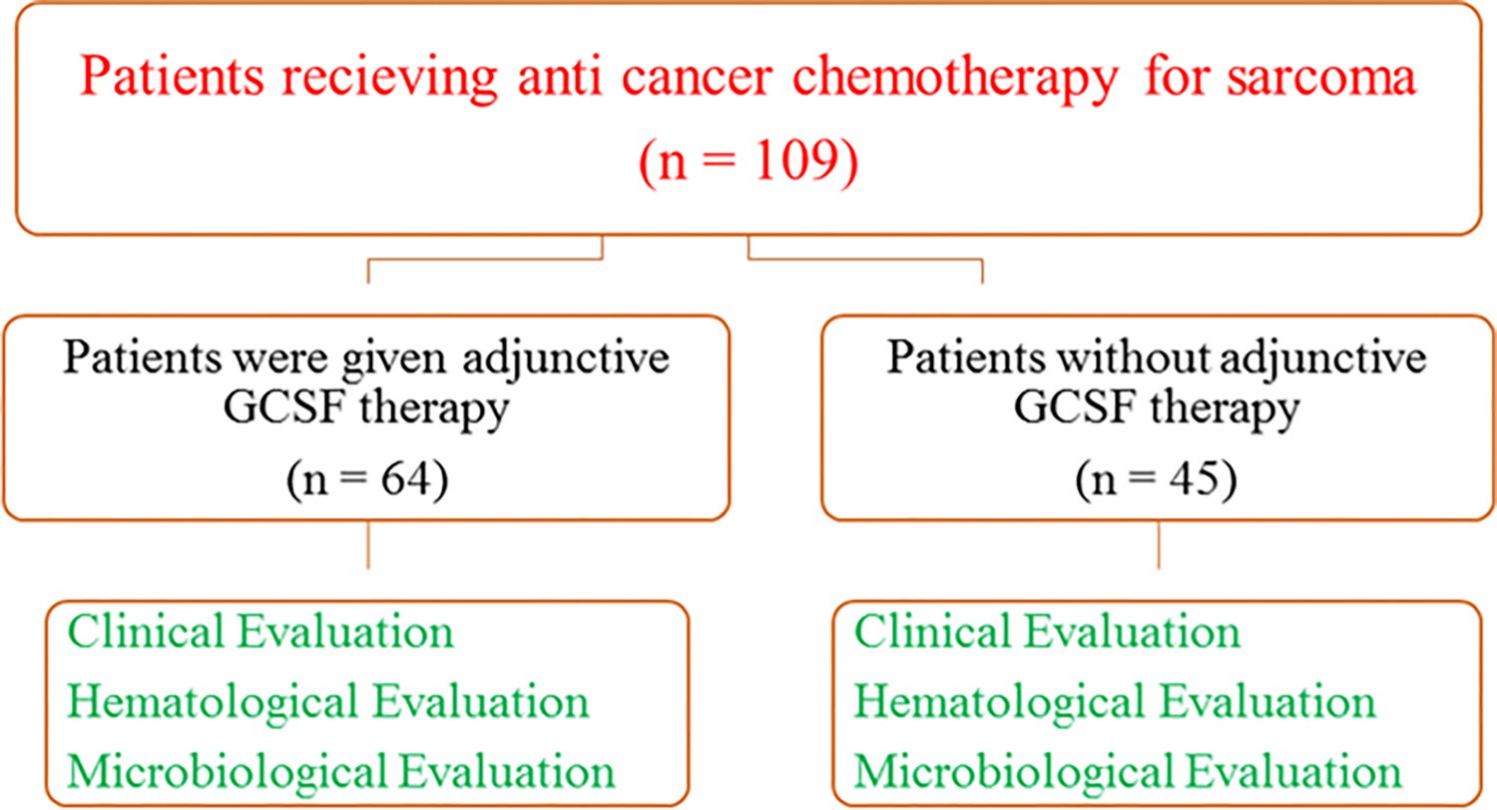
Schematic presentation of the study design.

All the patients were briefed about the study and a written consent form was signed by the patients or their attendant before inception of the study. Fig 1 shows schematic presentation of the study design.

### Patients’ inclusion and exclusion

Adult cancer patients of both the genders suffering from carcinom, getting anticancer chemotherapy and with developed FN, were included in the study. Furthermore, the definition of FN as mentioned by National Comprehensive Cancer Network (NCCN) guidelines was used for patients’ inclusion [15,16]. Prophylactic GCSF was prescribed in accordance to existing myeloid growth factor guidelines laid down by the American Society of Clinical Oncology (ASCO) and NCCN. GCSF is recommended for all patients getting high-risk FN chemotherapeutic regimens (>20%) and some patients receiving intermediate risk chemotherapeutic regimens (10–20%). Oncologist had the sole authority to prescribe adjunctive GCSF for treatment of FN. In our hospital, the use of adjunctive GCSF would commence within the first 24 h of chemotherapy induction.

Patients with multi organ failure, prophylactic antimicrobial therapy during initial 48 h of admission, fever due to blood transfusion or its components were excluded.

### Study procedures

Demographic (age, gender, and ethnicity) and medical information of the patients was collected by making a data collection form. Patients themselves, hospital information support system and the pharmacy prescription database were the sources of obtaining data. The clinical data consisted of;

- Medical history (cancer type, FN risk for anticancer treatment given, prior FN episodes and factors for developing FN) [17]
- Treatment record (chemotherapy, type and dose of G-CSF)
- Treatment outcomes (hospital stay because of FN, ANC recovery time, length of fever disappearence, and FN length)

Baseline hematological and biochemical tests were conducted at the designated hospital. Blood samples will be collected for culture sensitivity tests. Upon completing the treatment protocol, follow-up tests for both hematological and biochemical parameters were carried out to assess the treatment’s effectiveness [18]. The clinical safety of the treatment was evaluated by tracking any associated adverse effects.

Participants were randomly assigned to study groups based on the presence or absence of G-CSF, and their treatment responses were assessed. Before starting antimicrobial therapy during hospitalization, a baseline evaluation of hematological and biochemical parameters was conducted. This included measuring White Blood Cell (WBC) counts, Red Blood Cell (RBC) counts, Hematocrit (HCT), Mean Corpuscular Volume (MCV), Mean Corpuscular Hemoglobin (MCH), Mean Corpuscular Hemoglobin Concentration (MCHC), platelet count, and different types of white blood cells such as Neutrophils, Monocytes, Eosinophils, and Basophils. Additionally, parameters like Red Cell Distribution Width (RDW), Mean Platelet Volume (MPV), Platelet Distribution Width (PDW), Alkaline Phosphatase, Serum Glutamic Pyruvic Transaminase (SGPT), bilirubin, blood urea, and serum creatinine were measured to gather the necessary data [19]. Follow-up assessments were done after the completion of the antimicrobial and GCSF treatment, with both hematological and biochemical parameters reassessed after 5–7 days to evaluate the effects of the respective therapeutic intervention.

### Statistical analysis

Descriptive statistics for demographic and medical information were used. Independent sample t test was used for comparison of continuous variables. Odd ratios were calculated for statistically significant model variables. The *p* values were considered statistically significant when p <0.05. The statistical analysis was performed using Statistical Package for the Social Sciences (SPSS) version 20.

## Results and discussion

### Demographics and clinical characteristics

In the current study all the patients diagnosed for solid tumor (n = 120) during January-2022 to January-2023, were enrolled in the study. A total of 109 patients were included in the final study while 11 patients were excluded because of incomplete data. Data showed that the ratio of female patients (69/109; 63.3%) was higher than the male patients (40/109; 36.7%), as depicted in Table 1. Majority of the patients (97.6%) were Pakistani (Pashtun) and the remaining (2.4%) were Afghan national.

**Table 1:** Evaluation of prevalence of sarcomas in different age groups.

Age	Below 30	31–40	41–50	51–60	61& above
Male	15	8	8	7	2
Female	13	11	18	26	1
Median	16	36	45	55	64.5
Mode	25	38	45	58	61
Percentage	25.69	17.43	23.85	30.28	2.75
*p*-value	0.1	0.15	0.05	0.01	0.2
B-H value	0.05	0.12	0.16	0.18	0.2
OR	1.99	1.25	0.77	0.46	3.45

BH; Benjamini-Hochberg Value.

OR; Odd Ratio.

Average age of the patients was 43.9 years. In age groups less than 30 years old there were 28 patients including 15 male (13.76% of the total patients) and 13 were females (11.93% of the total patients). The age group of 31–40 years had 19 patients (8 male and 11female). Age group containing highest number of patients was 51–60 years (33/109; 30.28%) while age group of 61 and above had the lowest number of patients (3/109; 2.75%). In higher age group, patients are more susceptible for developing FN due to a fall in bone marrow reserve and decrease in immune function [20,21]. They need aggressive management and are benefitted more with prophylaxis of GCSF. Correlation of statistical parameters showed higher tendency of the age group 51–60 years for development of FN. The values of all the parameters (p value, odd ratio and BH value) pointed out to the higher likelihood of this age group as compared to the other age groups. Our findings were in line with the reported data [13–15] that chances of FN increases with the age.

Most of the patients were treated for Ca gastro (52/109; 47.71%) while the least number of patients was observed for thyroid cancer (2/109; 1.83%), as shown in Table 2.

**Table 2:** Ratio of male and female patients in different types of sarcoma.

Cancer Type	Total Patients (No.)	Total Patients (%)*	Male (No.)	Female (No.)	OR
Carcinoma Breast	31	28.44	0	31	0 .0
Carcinoma Stomach	18	16.51	10	8	2.1
Carcinoma Esophagus	4	3.67	0	4	0.0
Carcinoma Colon	13	11.93	6	7	1.48
Carcinoma Pancreas	5	4.59	3	2	2.59
Carcinoma Gall Bladder	3	2.75	1	2	0.86
Carcinoma Rectum	5	4.59	2	3	1.15
Secondary Tongue	4	3.67	2	2	1.72
Carcinoma Prostrate	7	6.42	7	0	Inf
Carcinoma Cervix	3	2.75	0	3	0.0
Carcinoma Ovary	5	4.59	0	5	0 .0
Carcinoma Lung	9	8.26	7	2	6.04
Carcinoma thyroid	2	1.83	2	0	Inf

OR; Odd Ratio.

*; Percentage was calculated on the basis of total number (n = 109) of patients enrolled in the study.

Initial clinical screening of the patients showed different co-morbidities as summarized in Table 3. Some of these comorbidities can be benefitted by the adjunctive GCSF therapy. Comparison of the results of comorbidities showed a significant treatment output with therapy containing GCSF. Bacterial infection was found in 85.32% of the patients while 11.01% patients had fungal infections. There is a notable reduction in bacterial infections among patients receiving GCSF therapy (8.22%) compared to those not receiving it (69.23%). This suggests that GCSF therapy might be associated with a reduced risk of bacterial infections. Recent studies indicated that GCSF can reduce the FN incidence and infections related to it by improving recovery of neutrophils and their function. GCSF reduces neutropenia duration, which in turn decreases the risk of bacterial infections [22]. The percentage of fungal infections appears to be higher in patients receiving GCSF therapy (2/12; 16.67%) compared to those not receiving it (0.00%). However, this is based on a very small sample size. Some studies suggested that GCSF may not significantly affect fungal infections [23,24]. The increased risk of fungal infections in neutropenic patients is more often related to the severity and duration of neutropenia rather than GCSF use alone [23]. Fever was significantly lower in GCSF treated patients (0%) compared to non-GCSF treated patients (39.13%) suggesting that GCSF therapy is effective in reducing fever (a sign of FN) by decreasing the duration of neutropenia and improving neutrophil function. This aligns with findings from studies that demonstrate reduced FN and associated symptoms with GCSF prophylaxis [25]. The incidence of pneumonia is higher among GCSF treated patients (50%), but this is based on only two cases. Pneumonia risk is generally linked to the severity of neutropenia and the overall health of the patient rather than GCSF use. The small number of cases in this dataset limits the ability to draw strong conclusions, but larger studies suggest that GCSF does not significantly increase pneumonia risk [21]. Hypotension is lower in GCSF treated patients (33.33%) compared to non-GCSF treated patients (100%).GCSF use is not typically associated with hypotension. The observed difference might be incidental or due to other factors. There is no strong evidence linking GCSF use with hypotension. The findings in this dataset might be influenced by the small sample size [26].

**Table 3:** Comparison of comorbidities resolution before and after therapy.

Comorbidity	Comorbidity before Therapy		Comorbidity with GCSF therapy		Comorbidity without GSCF therapy	
	Reported patients (No.)	Percentage (%)	Reported patients (No.)	Percentage (%)	Reported patients (No.)	Percentage (%)
Bacterial Infections	93/109	85.32	6/73	8.22	9/13	69.23
Fungal infection	12/109	11.01	2/12	16.67	0/0	–
Fever	109/109	100	0/86	0.00	9/23	39.13
Pneumonia	2/109	1.83	1/2	50.00	0/0	–
Hypotension	14/109	12.84	3/10	33.33	4/4	100

GCSF; Granulocytes Colony Stimulating Factor.

### Evaluation of FN

The primary parameter to evaluate outcomes of the study were the rate, duration and incidence rate of FN. Secondary endpoints were;

- Time to absolute neutrophil count (ANC) recovery
- Depth of ANC (lowest ANC)
- Time to neutrophil recovery (defined as the time from chemotherapy administration until the ANC increased to ≥2.0 × 109/L after the expected)
- Need for antibiotic prophylaxis or treatment.

### Baseline data of the patients taking adjunctive GCSF

A total of 109 patients completed the study out of which 64 (58.72%) received adjunctive GCSF therapy, and 45 (41.28%) did not receive adjunctive GCSF. Details of cancer type, chemotherapy regime and GCSF are listed in Table 4. Patients receiving GCSF therapy across various cancer types and chemotherapy regimens generally show a reduction in FN. While on the other hand FN was observed in all the patients not receiving GCSF. Numerous studies have demonstrated that GCSF reduces the incidence of FN by promoting faster recovery of neutrophil counts and reducing the duration of neutropenia [27]. High-intensity chemotherapy regimens (e.g., adriamycin and cyclophosphamide, doxorubicin, cisplatin, and paclitaxel) are associated with a higher FN risk. High-intensity regimens increase the risk of FN, and GCSF prophylaxis is particularly beneficial [25]. The efficacy of GCSF in reducing FN has been well-documented across a range of cancers. GCSF helps in preventing FN in patients undergoing high-risk chemotherapy regimens and has been shown statistically significant results in different clinical trials [21]. The reduction in FN with GCSF therapy has been observed with different chemotherapy regimens, suggesting that GCSF is effective across different drugs and their combinations. However, choice of GCSF dose and timing might vary based on the chemotherapy regimen and patient factors [26].

**Table 4:** GCSF with different anti-cancer chemotherapy regimens.

Cancer Type	Chemotherapy	Chemotherapy Intensity Level	GCSF	No. of Patients	FN
**Patients without adjunctive GCSF therapy**					
**Ca Breast**	Adriamycin and Cyclophosphamide	High	No	14	+ve
	Taxotere and Cyclophosphamide	Intermediate	No	4	
	5Fluorouracil, Adriamycin and Cytoxan	High	No	4	
	Carboplatin + Paclitaxel	Intermediate	No	9	
**Ca Prostrate**	Docetaxel	Intermediate	No	7	
**Ca Ovary**	Carboplatin and Taxol	Intermediate	No	5	
**Ca Thyroid**	Doxorubicin, Cisplatin, and Paclitaxel	High	No	2	
**Patients with adjunctive GCSF therapy**					
**Ca Stomach**	5- Fluorouracil and Oxaliplatin	Intermediate	Inj. Filgen (Filgrestim 300 μg) Single dose was administered after 24 hours of anti-cancer chemotherapy	18	–ve
**Ca Esophagus**	Capecitabin and Oxaliplatin	Intermediate		4	
**Ca Colon**	Folinic acid,5-Flourouracil and Oxaliplatin	Intermediate		13	
**Ca Pancreas**	*Capecitabin*,*Gemcitabin and Cisplatin*	High		3	
	5- Fluorouracil and Oxaliplatin	Intermediate		2	
**Ca Gall Bladder**	*Gemcitabine and Cisplatin*	High		3	
**Ca Rectum**	Capecitabin and Oxaliplatin	Intermediate		2	
	5-Fluorouracil and Leuvocorin	Intermediate		1	
	Folinic acid,5-Flourouracil and Oxaliplatin	Intermediate		2	
**Secondary Tongue**	5-Fluorouracil and Carboplatin	Intermediate		4	
**Ca Cervix**	Cisplatin and Paclitaxel	High		3	
**Adenocarcinoma lung**	*Gemcitabine and Cisplatin*	High		9	

Ca; Carcinoma.

+ve; FN is reported in patients.

–ve: FN was not reported in patients.

### Evaluation of Group-1 (GCSF given) patients

Prophylactic administration of GCSF is used to boost the production and function of neutrophils in cancer patients who are at high risk of developing FN due to chemotherapy [9,28]. GCSF primarily affects neutrophils but can also have some impact on other types of white blood cells (WBCs). G-CSF is specifically designed to stimulate the production and release of neutrophils from the bone marrow. As a result, it significantly increases neutrophil counts in the blood. While GCSF primarily targets neutrophils, it can also have some impact on other granulocytes like eosinophils and basophils, as well as monocytes. Eosinophil and basophil counts may decrease temporarily as more neutrophils are produced and released into the bloodstream. Monocyte counts may also increase slightly due to GCSF’s effects on the bone marrow. GCSF’s primary effect is on granulocytes (neutrophils, eosinophils, and basophils), and it typically does not have a significant impact on lymphocytes. In some cases, there may be a mild reduction in lymphocyte counts during GCSF treatment, but this effect is generally not as pronounced as the increase in neutrophils.

It’s important to note that the changes in blood cell counts seen with GCSF are generally well-tolerated and do not typically lead to clinical problems [29]. The main objective of GCSF is to reduce the risk of FN, which can be life-threatening in patients undergoing anti-cancer chemotherapy. The specific impact on blood cell counts can vary depending on individual patient factors, the dosing and duration of GCSF treatment, and the underlying cancer and chemotherapy regimen. Healthcare providers closely monitor these parameters during treatment to ensure the patient’s safety and the effectiveness of the prophylaxis.

GCSF primarily influences neutrophil counts and function, it does not have substantial effects on other blood cell types, including red blood cells and platelets. Any changes in MCH, MCV, RDW, HCT, MCHC, or MPV during G-CSF prophylaxis are likely related to factors other than the GCSF treatment itself, such as the patient’s overall health, underlying medical conditions, or the effects of chemotherapy.

FN did not develop in all the patients who received GCSF while on other hand, FN was observed in all the patients without GCSF. Neutrophil count of most of the patients remained within the normal limits with GCSF therapy. About 19% of patients had neutrophil count below the normal limit but still above the limit for FN and they had no sign and symptoms of FN. About 98% of the patients were infection free. Infection was observed in 2% of patients and both were infected with multi resistant bacteria (MRSA) [30]. In majority of the patients neutrophil count raised above the normal level and fever was not observed in any of the 98% patients.

Filgrastim (FIL; Neupogen®) got the approval of US-FDA in 1991 and has been used for the treatment of FN [26]. Different short acting GCSF products (like lenograstim and tbofilgrastim) and long acting products of GCSF (pegfilgrastim (PEG-F) and lipegfilgrastim) have been developed and are effectively used in different carcinomas [31]. Long acting GCSF has the advantage of avoiding regular injections are always preferred over short acting GCSF by the oncologists.

### Evaluation of Group-2 (without adjunctive GCSF therapy) patients

#### Hematological evaluation

Hematological evaluation included determination of RBCs, WBCs, platelet count, neutrophil count, HCT value, hemoglobin level, renal function tests and liver function test. All the parameters were determined as per established protocols and results were summarized in Table 5. In all the patients taking anti-cancer chemotherapy, rapidly growing cells were affected. Fewer patients with abnormal WBC counts suggests GCSF efficacy in managing and preventing FN. GCSF does not affect Hemoglobin, RBCs, HCT, and platelet count. These can be managed with other supportive therapies [32] and does not impact elevated liver enzymes as well as kidney function markers [33].

**Table 5:** FN treatment outcomes with and without adjunctive GCSF therapy.

Blood Test	References values of the blood test (Unit)	Patients with Normal Values (No.)	Patients with values below the limits		
			Total number of patients (No.)	With GCSF(No.)	Without GCSF (No.)
Hemoglobin	11.6–16 (g/dL)	94	6	1	5
Serum Bilirubin	0.3–1.0 (mg/dL)	23	23	4	19
WBCs	4.0–11.0 (10^3^/μL)	98	2	0	2
RBCs	3.80–5.30(10^6^/μL)	96	4	0	4
HCT	34–48 (%)	92	8	3	5
Platelet Count	150–450 (10^3^/μL)	97	3	0	3
Alkaline Phosphatase	Up to 258 (units/L)	94	6	0	6
SGPT	05 to 40 (units/L)	97	3	0	3
Blood Urea	15–50 (mg/dL)	98	2	0	2
Serum Creatinine	0.5–1.2 (mg/dL)	99	1	0	1

WBC; White Blood Cells.

RBCs; Red Blood Cells.

HCT; Hematocrit.

SGPT; Serum Glutamic Pyruvic Transaminase.

It was observed that the specific changes in complete blood count (CBC) results varied with the patient’s overall health, the severity of neutropenia, the type of infection, and the effectiveness of antibiotic therapy. The CBC is just one tool that healthcare providers use to monitor the progress of neutropenic cancer patients and their response to treatment. Details of hematological evaluation before and after therapy are presented in **Table 6**. It is indicated by results that WBC and neutrophil counts are improved by GCSF effectively, which is in accordance with its clinical use for FN prevention and management. Any change in other blood parameters may reflect the overall influence of cancer treatments and patient health rather than direct effects of GCSF [20].

**Table 6:** Hematological evaluation of neutropenic patients after treatment with GCSF alone.

Parameter	Baseline	Follow Up	Difference	Paired T Test
Hemoglobin	9.623	9.67	0.04675	0.876
White Blood Cells) Count	1.954	5.853	3.8985	0
Red Blood Cells Counts	3.355	4.67325	1.31825	0.003
Hematocrit	28.382	30.355	1.9725	0.078
Mean Corpuscular Volume	83.81	81.215	-2.595	0.015
Mean Corpuscular Hemoglobin	28.38	27.33	-1.05	0.11
Mean Corpuscular Hemoglobin Concentration	34.068	33.63	-0.4375	0.382
Platelet Count	150.761	270.62	119.85925	0.006
Neutrophils Count	24.204	36.0425	11.8385	0
Lymphocyte Count	26.726	16.78775	-9.93875	0.026
Monocytes Count	12.873	11.7875	-1.0855	0.707
Eosinophils Count	2.278	2.7055	0.42725	0.805
Basophils Count	0.177	1.211	1.034333	0.859
Red Cell Distribution Width	16.8025	18.4355	1.633	0.077
Mean Platelet Volume	7.32125	7.28925	-0.032	0.94
Alkaline Phosphatase	383.842	226.575	-157.26715	0.225
Serum Guanine Phosphate Transferase	52.6	33.0925	-19.5075	0.082
Bilirubin total	1.988	1.19725	-0.790184	0.214
Blood urea	33.62702703	16.665	-16.9627	0.001
Serum creatinine	0.724102564	0.44415	-0.27995	0

Along with hematological evaluation, the measured clinical outcomes showed that patients receiving adjunctive GCSF had better treatment outcomes, as compared to the patients without GCSF, as shown in **Table 7**. The benefits were clinically evident and patients showed significant improvement in symptoms like better control of fever, quick recovery in neutrophil count, decreased use of antibiotics, decrease in hospital stay, and resolution of infection.

**Table 7:** Treatment group with and without GCSF.

Treatment Group	Sample Size	Status	Mean	Standard deviation	Correlation	Significance	Paired t test
Chemotherapy with adjunctive GCSF	N = 45	Baseline	11.25	5.19	-0.014381	5.9495x10^-2^	1.9345
		Follow Up	9.5	4.09			
Chemotherapy without GCSF	N = 64	Baseline	16.0	11.01	0.006050	2.3908x10^-18^	12.2668
		Follow up	1.5	0.866			

#### Microbiological evaluation

FN presents a significant challenge and make the patients prone to the severe and life threatening infections. These infections can lead to increased hospitalization, necessitate antibiotic treatments, potentially reduce or delay crucial chemotherapy, and adversely affect quality of life. Pyrexia is a key indicator for diagnosing FN, as it often results from infections in patients with compromised immune defenses. In this study, every patient was assessed for microbial presence, with blood samples collected and analyzed for pathogenic organisms. Microbes were identified in all patients who did not receive GCSF (Group-2). The difference in microbial presence between male and female patients was not statistically significant (p = 0.09). Among the positive cases, 53.33% (24/45) were male, and 46.67% (21/45) were female, indicating a minimal gender difference in the presence of microbes. Notably, MRSA and Salmonella typhi were only found in patients who received GCSF, and none were detected in those who did not receive the treatment. All patients in the GCSF group with FN had these infections. Both MRSA and Salmonella typhi are known for their resistance to multiple antibiotics and their potential to cause serious infections in patients with weakened immune systems. The fact that these pathogens were exclusively isolated in patients receiving GCSF suggests that these individuals may have been more susceptible to such infections, possibly due to severe immunosuppression or changes in immune function following GCSF therapy. While GCSF stimulates the production of neutrophils and can increase their count, it does not fully restore immune function. Patients undergoing GCSF treatment may still be at high risk for infections from resistant pathogens if their overall immune system remains compromised. This finding is consistent with research indicating that while GCSF can reduce the incidence of FN, it does not entirely eliminate the risk of infections, particularly with resistant organisms [34].

**Fig 2:**
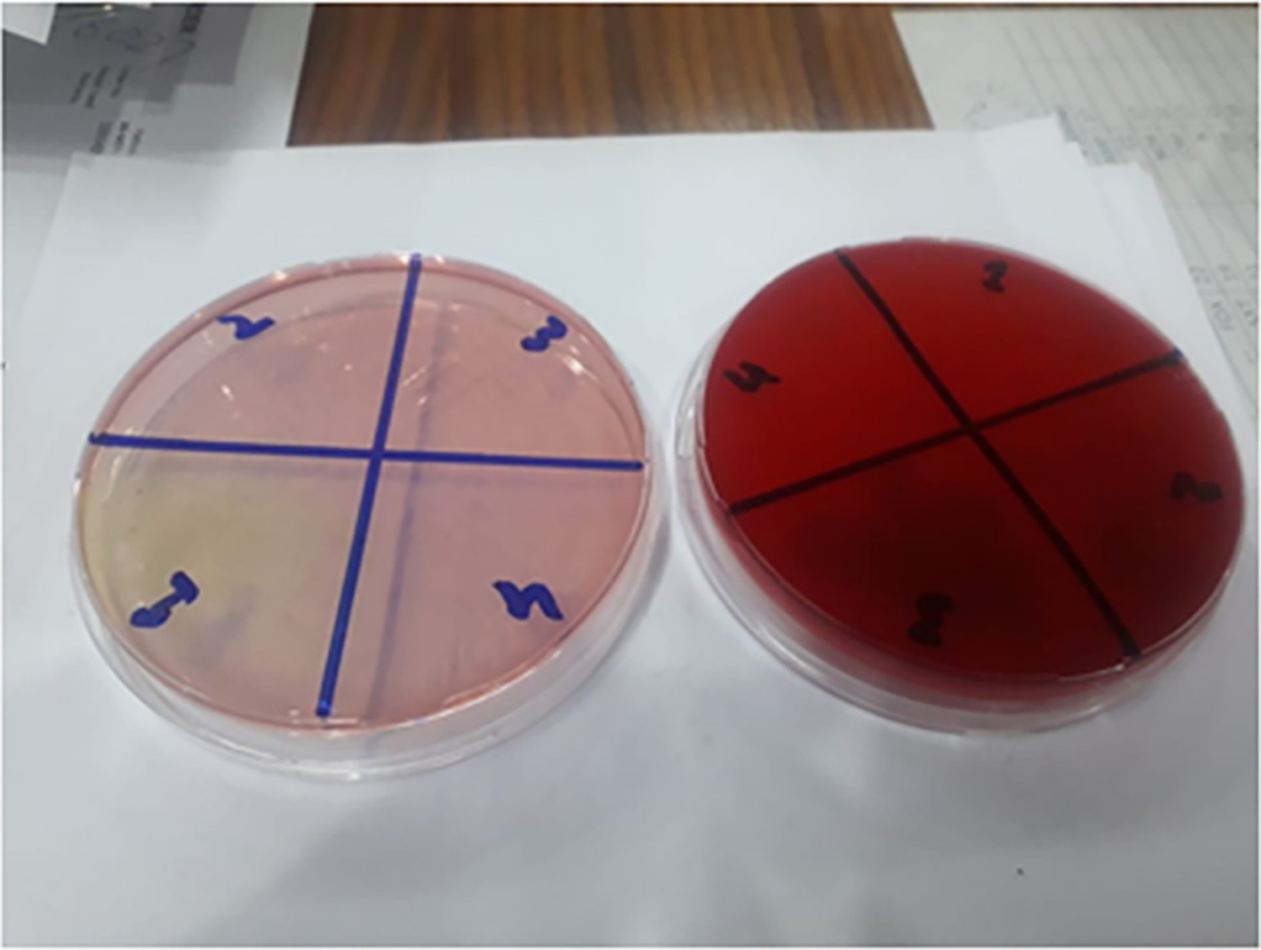
Cultures of blood and urine sample for determination of bacterial growth in the samples.

Both *E*. *coli* and *Klebsiella pneumoniae* were isolated only after non-GCSF therapy, with no cases reported in the GCSF group. The absence of these pathogens in the GCSF group could indicate that GCSF therapy might be associated with a lower incidence of these specific bacteria, possibly due to more effective management of neutropenia and reduced infection rates. Alternatively, the findings might reflect a smaller sample size or variations in the patient population. Research suggests that GCSF can help reduce the incidence of infections by managing neutropenia effectively. The lack of these bacteria in the GCSF group could reflect the therapy’s success in controlling neutropenia, thereby reducing susceptibility to common pathogens like E. coli and Klebsiella. However, comprehensive data are required to confirm this hypothesis [22]. Fig 2 shows images of blood culture and urine samples.

The mean value decreases from baseline to follow-up, indicating a reduction in the measured parameter. The correlation is close to zero which is very low, suggesting minimal relationship between baseline and follow-up values. The p-value (0.059495) is close to (0.05) suggesting a trend towards significance but not meeting it strictly. The t-value (1.9345) supports this trend but also indicates that the result is not statistically significant at the 0.05 level. This result indicates that GCSF treatment showed improvement, without any statistically significant change in this specific parameter. Studies generally support that GCSF is effective in reducing FN by stimulating neutrophil recovery. The trend here, while not statistically significant, aligns with evidence suggesting GCSF helps manage neutropenia, but the degree of impact can vary [35]. The noteworthy reduction in the mean value from baseline to follow-up for chemo without GCSF group suggests a large impact. The correlation is very low, indicating minimal relationship between baseline and follow-up values. The p-value (2.3908 x 10^−18^) is extremely small, showing a very significant outcome. The very high t-value (12.2668) additionally confirms that the difference is statistically significant. This indicating a strong treatment effect, with a substantial improvement in the measured parameter during the follow-up period. This result could reflect a situation where the lack of GCSF leads to more pronounced neutropenia or FN during chemotherapy. Research often shows that without GCSF, patients are at a higher risk of FN due to delayed neutrophil recovery. The significant change here underscores the effectiveness of GCSF in preventing FN and supporting neutrophil recovery [34].

## Conclusion

This study showed that adjunctive GCSF therapy has clinical benefits in patients with sarcoma. Along with hematological evaluation, the measured clinical outcomes showed that patients receiving adjunctive GCSF had better treatment outcomes, as compared to the patients without GCSF. The benefits were clinically evident and patients showed significant improvement in symptoms like better control of fever, quick recovery in neutrophil count, decreased use of antibiotics, decrease in hospital stay, and resolution of infection.
